# Medical Charities

**Published:** 1858-09

**Authors:** 


### MEDICAL CHARITIES.

During the present year several charitable medical institutions have been opened in this city.

A Dispensary has been maintained at the Medical College in the North Division, under the charge of Drs. Powell and Durham; and another at the corner of Clinton and Randolph sts. in the West Division, under the care of Drs. Wardner, Hollister and Andrews.

The Chicago Charitable Eye and Ear Infirmary has also been some months in operation, under the direction of the following officers and attendants:

*Trustees.*

| | |
|---|---|
| W. L. Newberry, *President.* | P. Carpenter. |
| Dr. C. V. Dyer, } *Vice Pres's.* | J. H. Kinzie. |
| L. Haven, } | E. B. McCagg. |
| S. Stone, *Sec. and Treasurer.* | F. Moseley. |
| Rev. W. Barry. | Rev. Dr. N. L. Rice. |
| W. H. Brown. | M. Skinner. |

*Consulting Surgeons.*

Prof. D. Brainard, M.D. Prof. J. W. Freer, M.D.

*Visiting Surgeons.*

Dr. E. L. Holmes. Dr. F. B. Norcom. Dr. H. Parker.

All the medical men connected with these institutions are in good standing in the profession, and entitled to the confidence of the community.

